# Modern Aspects of the Complex Interactions between Biodiversity and the Main Neuropsychiatric Disorders

**DOI:** 10.3390/brainsci13081205

**Published:** 2023-08-15

**Authors:** Madalina Borca, Alin Ciobica, Alin Iordache

**Affiliations:** 1 Department of Biology, Faculty of Biology, Alexandru Ioan Cuza University, B dul Carol I, No. 11, 700506 Iasi, Romania; 2 Center of Biomedical Research, Romanian Academy, B dul Carol I, No. 8, 700505 Iasi, Romania; 3 Academy of Romanian Scientists, Splaiul Independentei 54, Sector 5, 050094 Bucuresti, Romania; 4 Department of Neurology, Grigore T. Popa University of Medicine and Pharmacy, 16, Universitatii Street, 700115 Iasi, Romania

**Keywords:** neuroscience, biodiversity, psychopathologies, biophilia, human ecology

## Abstract

The high incidence of psychopathologies recorded in today’s human society, correlated with the high percentages of biodiversity loss, point to the need for an interdisciplinary approach of the scientific fields under study—neuroscience and biodiversity conservation. Thus, our approach here presents, in a synergistic manner, the significant correlation between mental health and the increased values of biodiversity in the ecosystems located in the immediate vicinity, especially those located in the middle of cities. Our approach aims to emphasize the importance of biodiversity conservation in the context of preserving mental health and general well-being. There are a series of recent experimental demonstrations that outline the influence of natural elements on the human psyche and, implicitly, the effects of nature in the prevention and reduction of stress, anxiety, and depression. And beyond the cognitive barriers of humanity in relating to the surrounding biodiversity must lie the desire to know the values of biodiversity and the absolute importance of its conservation. The sustainable relationship between humans and living nature, seen as a complex of biodiversity, is dealt with by a branch of science called human ecology. Therefore, this study emphasizes the crucial need to know and respect the connection between man and nature, based, since time immemorial, on biophilia. And with the regression of ignorance and the correlated approach of several scientific fields, some at the intersection of the humanities and natural sciences, one can observe the progress of preserving the dynamic balance within ecosystems and, implicitly, the preservation of mental health and human well-being.

## 1. Introduction

The point of convergence of two distinct scientific fields—which our analysis of previously published studies approaches in an interdisciplinary manner—biodiversity and neuroscience, can be found in the notion called biophilia, denoting the impulse to affiliate with other forms of life. The innate tendency of human beings to focus their interest on life and vital processes is a scientific hypothesis attributed to adaptive learning in phylogeny, characterized by fascination and asymmetric empathy [1]. The term biodiversity encompasses all the organisms present in the various living environments, including terrestrial, marine, and other types of ecosystems associated with the ecological complexes of which they are a part [2]. An ecosystem represents a complex group of dynamic organisms that function as a unitary whole. The mosaic of ecosystems, the species that live in various habitats, and the genetic variability within each population synergistically define the notion of biodiversity [3]. In reference to human health, and particularly to mental health, the relationship between biodiversity and neuroscience can be highlighted by the occurrence of psychopathologies in situations where biodiversity decreases, but also by the high quality of life lived in urban environments with high rates of biodiversity. The acronym NCP (Nature’s Contribution to People) describes ecosystem services, that is, the direct and indirect contributions of nature to human existence and well-being.

Ecosystem services include supply, regulation, and cultural services. Therefore, human beings are in permanent dependence on the natural environment in which they live, in a privileged position, and even “on the shoulders of nature” [4]. This study emphasizes the need to understand the mutualistic relationship between mental health associated with human well-being and the constant need to conserve and protect the biodiversity of the surrounding environments. Therefore, in the following sections, we shall explain how the relationship between the aesthetic value of landscapes and biodiversity has scientific relevance in the context of human health, but also in the context of conservation biology.

**Figure 1:**
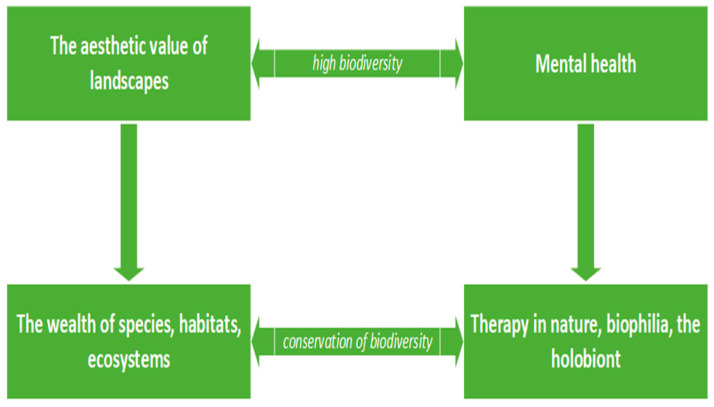
Schematic representation of the interdisciplinary study, including the basic notions and the connections between them.

Also, Figure 1 presents a basic representation of the interdisciplinary study, including the basic notions and the connections between them. One can observe the links between the fields studied and the complexity of the interaction generated by the high values of biodiversity related to the importance of the mental health of contemporary humans. The aesthetic function of ecosystems takes various forms due to the abundance of species that compose it, and in its turn the ecosystem serves as a “protective umbrella” against psychopathologies that are increasingly present in today’s society. Note that therapy in nature, based on biophilia, may represent a means of psychological rehabilitation.

## 2. Methodology

The studies were searched in databases containing scientific articles (e.g., ScienceDirect and Google Scholar). We used the following keywords: ‘neuroscience’, ‘biodiversity’, ‘psychopathologies’, ‘biophilia’, ‘ecosystem services’, ‘climate change’, ‘nature therapy’, ‘ecoanxiety and solastalgia’, and ‘biodiversity and mental health’. The selection process was conducted regardless of the articles’ publication date and included articles up to July 2023. Firstly, the publications were screened by title, then by abstract content, and then by full content. We approached the studies cited in an interdisciplinary manner in order to highlight the link between psychopathologies and biodiversity, emphasizing the need to preserve biodiversity as a method of prophylaxis within human neuropsychopathologies.

## 3. High Biodiversity—A Defining Factor of Human Well-Being

Viewed from the perspective of biophilic theories, biodiversity, perceived by most people in the form of landscape aesthetics, actually represents a direct source of health due to the emotional affinity of humanity towards the rest of living organisms [5]. However, a prospective analysis of recorded data is needed in order to understand the correlation between biodiversity and mental health [6]. The reason is that people’s perception depends on several external factors, such as cultural factors and religion, the urban or rural environments in which the subjects have lived, and also the idealistic preconception of a compact nature, viewed as a landscape rather than as a dynamic complex of habitats that are biologically diverse [7].

Regarding the significant correlation between human well-being and biodiversity, a number of emotional changes were identified following exposure to green spaces with a high biodiversity: stress reduction, improvement in cognitive function (including memory and attention capacity), considerable increases in social interaction, improvement in academic abilities and self-esteem, as well as increased creativity inspired by natural settings [8].

Short-term exposure to forests, urban parks, gardens, and other natural environments reduces stress and depressive symptoms, reduces fatigue, increases the ability to focus attention, increases positive emotions, and engenders an optimistic mood [9].

There are important studies that analyze the positive effects of biodiversity on human well-being and studies focusing on humans’ interactions with green spaces in their immediate vicinity or even in the middle of cities. It has been shown that various natural settings can generate distinct psychological restoration effects, but the relationship between biodiversity levels and the feeling of well-being is still ambiguous and controversial. At the same time, it is necessary to investigate the mechanisms underlying the relationship between the richness of biodiversity and positive psychological responses, as well as the categories of moderators of the relationship described previously [10].

Another approach, One Health, correlates the beauty of nature and the connections that can be made with it using an experimental study involving 30 days in the wilderness [11]. Thus, it was established later, following the study, that there is a correlation between the beauty of nature and the feeling of well-being, a correlation based predominantly on the human sensitivity towards the beauty of natural settings [11]. Regarding the perception of biodiversity, the ability to identify component species in natural intra-urban ecosystems, and the relationships between biodiversity and well-being, there is a link between the feeling of well-being and the level of biodiversity provided by green spaces in cities; positive emotions and even recovery after mental fatigue result from the interaction [12].

Such new assessments of exposure and response to environmental factors are essential for understanding biodiversity-based therapies and for informing environmental policies that seek to maintain and develop nature that is beneficial to human health inside and outside urban areas, such as solutions based on wildlife and green infrastructure [13].

## 4. Holobiont—The Defining Link between Intra-Urban Biodiversity and Human Health (in the Broad Sense)

For the correct approach to this type of scientific concept, the symbiosis between organisms must be seen as a source of evolutionary innovation, and ecosystem services must also be understood down to the level of the microbiota in the living environment.

The notion of holobiont means both the host and its microbiota, which together constitute the metagenome subjected to the pressure of natural selection [14].

The bidirectional MGB axis (microbiota–intestine–brain axis) represents the direct communication between the brain and the gut microbiota, and preclinical studies have shown that dysbiosis is a factor of interest in anxiety, stress, and mood disorders [15].

It is known that people who live in cities or urbanized areas with increased biodiversity get sick less often than those who live in the big urban metropolises of the Globe, and the explanation is simple: the presence of natural settings and of the most diverse green spaces gives them a high immunity and a state of well-being that supports this immunity [16].

## 5. Biodiversity and Mental Health

In order to be able to express the importance of biodiversity for the preservation of mental health, but also for the prevention of psychopathologies, we shall use the concept of *nature experience*, which describes the perception of each individual in relation to the natural environment that surrounds him/her [17]. This space may be an urban garden or a park, a mountain trail in a nature reserve, or any other natural setting in which the subject connects directly—by means of the SNS (somatic nervous system), which integrates the body into the living environment—to the surrounding nature, which basically includes the biodiversity of the habitats perceived with the help of the sensory organs (visual, auditory, gustatory, olfactory, tactile, etc.). The perceptions of the sensory organs can be based either on reality or on experimental simulations (e.g., using photographs or even virtual reality) [18].

Humans are multisensorial beings, and multiple studies demonstrate the fact that the senses are interdependent, informing each other about stimuli from the external environment [19,20]. Regarding the visual sense, research has shown that viewing even a small number of indoor plants can increase pain tolerance [21]. Similarly, looking out the window upon green spaces in the vicinity is used for patients in hospital environments as a means of recovery, as this reduces perceived pain and engenders a state of well-being [22,23]. The visual means through which emotional well-being is restored are based on two theories with evolutionary origins. The first is the theory of stress reduction, also known as the psychoevolutionary theory formulated by Ulrich, which is strongly associated with biophilia [24]. The second theory is the attention-restoration theory, which claims that people focus better after spending time in nature or visualizing natural settings [25]. About the auditory sense, it is claimed that there is a neurological correlation between it and the visual sense [26,27]. Sounds are context-dependent, from which it follows that the seasons, for example, can shape perceptions [28,29]. The neurobiological theory states that interactions between individuals and their living environments have an impact on brain activity, enriching motor and sensory perceptions [30].

Specialized studies conducted on the topic of the correlated link between biodiversity and mental health estimate a reduction in anxiety, depression, and chronic stress relative to high values of biological diversity [31]. Another notable example is the one that uses, in an experimental study, smartphones to monitor the well-being of the participants in real time; the results point out that even short-term exposure to natural elements has important beneficial effects on mental well-being, and that the lasting impact of a well-installed condition can already be detected a few hours after exposure to the elements of nature [32].

The concept of neurodiversity describes the normal differences of the human brain that determine characteristic ethology related to the environmental conditions with which each individual interacts. In the following paragraphs, we shall present some clear examples of individuals with symptoms of ADHD (attention-deficit hyperactivity disorder) and autism who, after interacting with elements of the surrounding nature, modify their perceptions of the living environment.

Worldwide, there is an estimate that approximately 7.2% of children have attention-deficit hyperactivity disorder [33]. A number of studies attest to the fact that exposure to natural environments may have protective effects against ADHD symptoms or may moderate the intensity of this psychopathology in the case of children [34,35,36].

Another study, carried out in New Zealand, demonstrates that rural environments and an increase in the area of green spaces in the community were strongly and independently associated with a reduced risk of ADHD symptoms among children, and that these elements of the natural environment could provide the highest rate of success in disease prevention [37].

Another pilot study, based on the therapeutic effects of gardening, presents the potential of caring for local biodiversity as part of the therapy of a target group of young people with autism. A significant improvement of the effectiveness of the rehabilitation objectives in patients with neuropsychiatric conditions is noted here, especially the improvement of social skills and interpersonal relations [38].

## 6. Dramatic Loss of Biodiversity and the Emergence of Psychopathologies

The consequences of the loss of biodiversity are, at present, also a real reason for concern from the point of view of the occurrence of psychopathologies among the human population. Along with the reduction of ecological integrity, the depletion of habitats, and drastic climate change, the states of anxiety, frustration, and depression have also increased [39]. Sometimes, climate change results in mass migration phenomena, which demonstrates, at the same time, the adaptability and ethological changes that occur in the case of these human populations. There are intersections between global climate change and mental health that have long been underappreciated. However, they can affect mental health in direct ways, such as the effects of natural disasters and extreme weather events (heatwaves, floods, drought, etc.), but also indirectly, such as the increase in migration rates and the existential inequity related to standards of living [40].

The vulnerability of habitats and the rapid extinction of their component species are caused by industrialization and urbanization, but also by mankind’s ignorance, which is why it is imperative to promote conservation biology, not just mental health, since an interdisciplinary approach can act synergistically to improve the relationship between humans and the surrounding nature.

Wild intra-urban nature could be a saving solution for the concrete implementation of the principle of sustainable development in the context of crowded and industrialized cities. This aspect involves an interconnected approach between the intelligent use of land, ecosystem services, and urban design in order to reconnect people with the natural environments in their immediate vicinity, with wild nature being seen as a corridor of biodiversity in the heart of the city. Any interaction with wild nature inside the city, once it has become a routine activity, is sufficient to ensure well-being and promote mental health while maintaining a high degree of urban biodiversity. Therefore, at the level of urban infrastructure, the implementation of green spaces is not enough when it comes to human well-being and, implicitly, mental health, and we must resort to the aesthetic values of living, biodiverse ecosystems [41].

## 7. Perspectives for the Future

Current research has demonstrated the fact that humanity adapts relatively slowly to climate change caused by ecological disasters, themselves attributable to human activity. Therefore, the accelerated loss of habitats that leads to the extinction of many species could cause a substantial reduction in people’s resistance to major environmental stressors. Since 2007, studies focused on this issue of human mental health positioned in the context of biodiversity loss have demonstrated the existence of a form of suffering, principally of an emotional nature, due to the awareness that contemporary humanity is facing drastic changes in the ecology of global bioclimatic zones. These so-called psychoterratic syndromes, which also include eco-anxiety and solastalgia, are such forms of challenges [42]. Essentially, through these manifestations attributed to ecological anxiety, the future of the following generations is questioned in contexts where the concept of sustainable development often remains only a theoretical concept, difficult to apply in the everyday life of modern humans. Ecopsychology is a scientific field consisting of numerous perspective categories that coexist and act synergistically, such as human ecology, psychology, philosophy, and spirituality, as well as conservation biology, viewed from the aspect of responsible environmental activism [43].

According to Alexandru N. Stermin, human ecology is the science that explores the dance between man and nature and the manner in which, over time, nature has shaped man and man has shaped nature. Nowadays, human ecology is the horizon and the potential source for solutions for restoring our relationship with nature and becoming friends with it [44].

Human ecology was born at the intersection of humanistic and natural sciences with the aim of emphasizing the coevolutionary relations between human society throughout its existence and its living environments, which are in continuous change. As an interdisciplinary field, it works in scientific symbiosis with other branches of biology, ecology, physics, medicine, anthropology, etc. [45]. Another discipline related to human ecology is ecosociology, which places *Homo sapiens sapiens* at the top of the food chain as a superior being from the point of view of its characteristic ethology and sociability.

Human ecology tries to restore the ancestral links between man and nature, emphasizing the importance of grounded knowledge of the ecosystem services that improve our existence on Earth. The concept of ecopsychology is used in a similar sense, and strategically points out the imperative need for therapy in nature and, at the same time, the need to preserve biological diversity and to promote concretely sustainable development aimed at ensuring the well-being and existential stability of future generations [46].

The aspect most worthy of being taken into account emerging from our analyses carried out from an interdisciplinary perspective is the fact that these far-reaching scientific fields—newly described in order to explain the speed of degeneration of the human psyche together with the reduction or even loss of biodiversity under the effect of various stressors, especially ecological ones—require enhancements in the context of clarifying the convergence of notions and phenomena described previously.

Still, it is believed that it will not be long, however, until scientific progress will explain more precisely what the experimental models and the hypotheses presented above have demonstrated so far. Our small study here emphasizes especially the need to expand these analyses and, at the same time, aims to expand the way in which most people relate to their surrounding natural settings.

**Figure 2:**
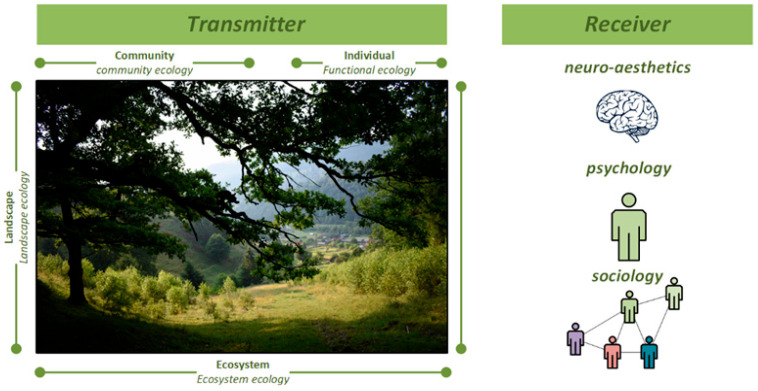
The interdisciplinary link between biodiversity and neuroscience.

In Figure 2, we present the complex forms of relationships that can be found between the elements that make up the term biodiversity, viewed as a whole, of which we recall the richness and abundance of species; the functional features of species alongside their structural, ecosystemic, and genetic diversity; and the preservation of mental health. Thus, in this bilateral communication relationship, nature, which consists of the surrounding biodiversity, receives plays a role as the transmitter of well-being, while the human community, integrated in the living environment, receives the transmitted message.

**Figure 3:**
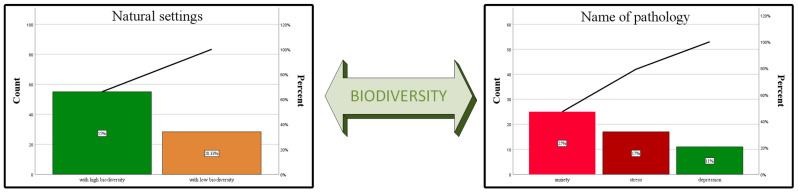
The interdisciplinary link between biodiversity, the aesthetic value of landscapes with high biological diversity (expressed in percentages), and the percentage values of the reduction of psychopathologies in relation to increased values of the surrounding biodiversity [23,47].

Figure 3 also depicts in a synthetic manner the significant interdisciplinary aspects, based on two distinct studies [23,47]. The chart related to natural settings shows in percentage values the fact that a landscape with a high species richness is much more complex, both from an ecological point of view and from the point of view of the aesthetics perceived by the human brain. Meanwhile, the chart related to psychopathologies shows the percentages by which they are diminished when the subjects are exposed to experiences in nature. Thus, did tried to make a connection between the two studies in order to emphasize the importance of biodiversity in the context of the prevention and mitigation of psychopathologies that occur increasingly more often in today’s human society.

There are, of course, several limitations to our approach here. The most important is related to the general lack of studies in this area. Of course, characterizing some quite heterogenous neuropsychiatric disorders in the context of biodiversity makes things even more complicated and the topic a little too broad to approach in a short opinion piece. However, we do consider that it is important to raise awareness on this important matter, and we do hope that our report will successfully achieve that.

## 8. Conclusions

An interdisciplinary approach is needed for the significant correlation between neuroscience and biodiversity conservation to be understood thoroughly within today’s human society. Thus, at the crossroads between the multiple scientific disciplines that our study mentions, we find the desire to emphasize the correct understanding of the overwhelming role that biodiversity has in the context of people’s mental health. Thus far, data have been presented regarding the need to integrate biodiversity, as unspoiled as possible, in urban spaces. These statistical data are based both on experiments using virtual reality and in vivo experiments testing the effects of natural settings on the human psyche. Based on biophilia and on the ability to adapt along phylogeny, all the sciences related to human ecology emphasize the crucial need for interdisciplinary knowledge of the complexity of biological diversity and of neuroscience. Our study also wishes to emphasize the need to expand interdisciplinary studies that aim to present the direct link between high biodiversity and mental health, but also the psychosocial balance of contemporary humans and mitigating psychopathologies, and that aim to provide prophylaxis in the case of psychopathologies simultaneously with the preservation of many ecosystems and habitats prone to mass extinction. However, it is still difficult to explain the primordial reality existing between elements of food chains in nature, where humans are both the cause and effect of global biodiversity loss. Therefore, more interdisciplinary studies are needed to emphasize that the loss of specific diversity leads to the early-onset of psychopathologies among contemporary people. Referring to the increased incidence of neuropsychiatric diseases caused directly or indirectly by the extinction of many species in anthropogenic ecosystems, it is still difficult to explain this degenerative process. Biophilia explains the ancestral connection of humans with nature, but contemporary humans and the complexity of their lives degrade this connection, making innovative studies necessary to clarify the role of biodiversity in maintaining mental health and general human well-being.

## Data Availability

All data is available on request.
